# Mothers’ Perceptions and Attitudes towards Children’s Vegetable Consumption—A Qualitative, Cross-cultural Study of Chilean, Chinese and American Mothers Living in Northern California

**DOI:** 10.3390/foods10030519

**Published:** 2021-03-02

**Authors:** Karinna Estay, Amalie Kurzer, Jean-Xavier Guinard

**Affiliations:** 1 Department of Food Science and Technology, University of California Davis, 392 Old Davis Road, Davis, CA 95616, USA; kestay@ucdavis.edu (K.E.); abkurzer@ucdavis.edu (A.K.); 2 Departamento de Agroindustria y Enología, Facultad de Ciencias Agronómicas, Universidad de Chile, La Pintana 11315, Chile

**Keywords:** feeding practices, eating behavior, children, mothers, vegetables, cross-cultural

## Abstract

This exploratory research focused on the cultural variables involved in children’s vegetable consumption, through the analysis of mothers’ perceptions, attitudes, and feeding practices regarding their children’s intake, using qualitative consumer research methods. Twelve focus groups of mothers with children between 2–12 years old (Euro-Americans *n* = 20, Chinese *n* = 19, and Chilean *n* = 19) were conducted. All participants lived in Northern California, had higher education, and incomes that did not limit their vegetable purchase. Intercultural differences in vegetable preferences and consumption habits were found. Mothers across all groups agreed on the importance of children’s vegetable consumption, the influence that mothers have over their children’s vegetable intake, and how challenging it is to get children to eat a variety of vegetables. The ethnic groups differed regarding how they perceived the level of mothers’ responsibility over children’s vegetable intake, the way that mothers defined the amount of vegetables that children should eat, the constraints that mothers had on increasing their children’s vegetable intake and mothers’ recommendations to encourage vegetable consumption. Our study suggests that under similar socio-economic and parental education levels, culture-specific strategies should be considered to foster healthy dietary habits in children.

## 1. Introduction

Children’s low vegetable consumption is an important public health concern [1,2,3]. This is because low vegetable consumption is a modifiable risk factor that is contributing to rising global mortality around the world [4,5,6]. Particularly, researchers have observed that low vegetable consumption increases the risk of the development of obesity, Type 2 diabetes, ischemic stroke, and some cancers [2,7,8,9,10,11]. Considering the health implications of low vegetable consumption, researchers have shown that by increasing children’s vegetable intake, it is possible to improve children’s health and their future health during adulthood [7,12]. Moreover, vegetables are characterized as a nutrient dense food, with low energy content, providing high nutritional value with low caloric intake [13,14]. The World Health Organization (2020) suggests the increase of vegetable consumption within individual strategies to reduce overweight and obesity. However, the innate factors relating to food preferences do not favor the liking and thus the intake of vegetables in children [13]. Humans are born with an innate preference for sweet taste and an innate rejection of bitter taste. In this regard, the sensory profile of vegetables, in conjunction with human’s innate food preferences, allows us to explain why vegetable acceptance needs to be developed through repeated experience with foods [14,15], and childhood is a critical time for the development of vegetable preferences [16,17,18]. This is mainly because eating behaviors established in childhood tend to persist later in life [16,17,19] and early vegetable consumption maximizes the health benefits associated with vegetable intake [18]. Moreover, intervention studies with children aimed to promote vegetable consumption have determined that younger children seem more likely to change their food behaviors than older ones [20].

Many factors influence the development of children’s food preferences, and among them, the food environment that parents provide during childhood is key to shaping children’s food preferences and their eating habits [18]. Parents influence children’s vegetable preferences through multiple conscious and unconscious mechanisms, through the food choices provided, as well as the feeding practices and the modeling effect that they exert on their children [21,22,23]. Researchers have shown that children tend to prefer and eat food to which they have been routinely exposed [24,25]. Families and specifically parents who eat more vegetables tend to have children with higher vegetable consumption [26,27]. Family influence does not only affect specific food consumption, but also the development of eating behavior. Branen and Fletcher (1999) reported that young adult eating habits were correspondent with the eating practices that their parents had during their childhood [28]. Even if parents are aware that their feeding practices exert a major influence on children’s food preferences and intake, these influences are not necessarily in the ways that parents expect. In this regard, researchers have shown that the use of pressure and/or reward for eating a disliked food, can produce an intake of this food in a short period of time; however, in the long term, such practices tend to reduce the preferences for the food being encouraged [26], and vegetables are among the foods that parents tend to force the consumption of through the previously mentioned practices [29]. In particular, mothers are not only responsible for feeding their children, they are also the earliest influencers of children’s food preferences, as children experience different flavors according to the mother’s diet in utero and later through breast milk [30]. In this sense, mothers’ beliefs, experiences, and visions of children’s vegetable consumption can provide critical information, useful for consideration in the promotion of children vegetable intake. 

This exploratory study aimed to analyze the perceptions and attitudes regarding their children’s vegetable consumption of Chilean, Chinese, and Euro-American mothers living in Northern California.

The specific objectives of this study were:

1. To explore mothers’ experiences and practices regarding their children’s vegetable consumption.
2. To explore the barriers to vegetable consumption and possible strategies for promoting vegetable consumption.
3. To analyze different cultural approaches to children’s eating habits and how they can influence children’s vegetable intake.

## 2. Materials and Methods 

### 2.1. Focus Groups 

A total of twelve focus groups sessions (four per ethnic group) were conducted with mothers in Northern California, following practices outlined by Casey and Krueger (1994) [31]. Sessions lasted between one hour and one and a half hours and were conducted within a two-month period. On average, each session was attended by five participants (typically between four and six). Mothers participated without their children, with the exception of two Chilean mothers who participated with their newborn sibling. Sessions were conducted in a focus group facility, equipped with a large table and one-way mirror, located in The Robert Mondavi Institute for Wine and Food Science at UC Davis, with the exception of the last Chilean focus group session that was conducted in Berkeley, CA, under similar conditions.

The team that conducted the focus groups was composed of the lead author, a moderator, and a research assistant. Considering the cross-cultural nature of this study, the moderators and the research assistants were specific for each ethnic group. Specifically, the first language of the moderators and the research assistants was Chilean Spanish (for the Chilean focus groups), Mandarin (for the Chinese focus groups), and American English (for the American focus groups). The moderators and research assistants for Chilean and Chinese focus groups were also fluent in English, allowing moderators and research assistants to participate in the transcription and translation process of the focus groups comments. The moderators who facilitated the focus groups sessions were all mothers, 28–33 years of age, each one of them belonging to one of the cultural groups studied: Chilean, Chinese, and Euro-American. All sessions were conducted in the first language of the participants: Chilean-Spanish, Mandarin, and American-English. All moderators and research assistants were trained in the conduct of focus groups. In order to maintain similar conditions, each moderator moderated the four sessions of each cultural group, and each research assistant participated in all the focus group sessions for their respective cultural group. Moreover, the first author was present in the twelve focus group sessions. In all sessions, a research assistant and the investigator were present on the other side of the mirror, analyzing the group dynamics, non-verbal expressions, and taking notes about the key information obtained in the session. All focus group sessions were audio- and video-recorded for subsequent reference and analysis.

The focus group sessions started with general instructions and a warm-up activity, then followed the script of questions (Table 1). The script was designed by the authors and vetted by a group of sensory and consumer scientists. It was designed to allow for the exploration of the research questions in this study while being consistent in the questions asked across cultural groups and sessions. 

In order to unify concepts among participants, during the introduction the moderator read the definition of vegetables for the purpose of this study: “Vegetables usually include different edible parts of a plant, like stems (ex: celery), leaves (ex: lettuce), buds (ex: cauliflower and broccoli), tubers (ex: potatoes), and roots (ex: carrots). From a botanical point of view, tomatoes, squash, and bell pepper are fruits; however, for the purposes of this study, we will call them vegetables, due to the way that we eat them. Just to be clear, today we are not going to include dry legumes in our definition of vegetables”. The moderators took a few minutes to ensure that the definition was understood by all participants. After that, the moderator followed the script, putting special emphasis on allowing a flow of ideas and opinions to be generated across participants, and also giving all participants the opportunity to join in the discussion. Finally, at the end of each session, a paper questionnaire was given to each participant in her first language to capture demographic information. The answers to these questions are reported in Table 2. 

### 2.2. Focus Group Participants

Fifty-eight mothers living in Northern California were recruited to participate in this study. Quota sampling was used to ensure a similar number of participants in each cultural group (19 Chilean, 19 Chinese, and 20 Americans). They were recruited through the University of California, Davis, networks by email and by personal invitations and/or flyers in Davis’ parent circles. For the Chilean participants, three groups were recruited in Davis, and the fourth group was recruited in Berkeley (California), though the UC-Berkeley Chilean Association. Participants were eligible based on: their self-identification into one of the three ethnic groups (Chilean, Chinese, and Euro-American/White Caucasian American), whether they were born in the country for which they were selected (Chile, China, and the USA), and if they were the mother of a child or children in the 2–12 years age range. The native language of the participants was Chilean Spanish (Chilean participants), Mandarin (Chinese participants), and American English (Euro-American participants). In total, the participants had 75 children in the 2–12 years age range (CL:24, CN:24, and USA:27). Before starting each focus group session, participants signed a consent form in which they agreed to the use of video cameras and tape recorders in the session. All participants completed the study and received a $20 gift cards for their participation. This study was approved for the use of human subjects by the Institutional Review Board of the University of California, Davis (IRB ID: 930546-1). 

### 2.3. Data Processing and Analysis

Transcriptions of the recordings and translations to English (for the Chilean and Chinese groups) were verified through a dual analysis process to guarantee the accuracy of the analyses. This process involved native speakers among the researchers and the moderators of the focus group sessions.

The data was analyzed through a careful reading and re-reading of the transcripts and of the notes taken during the sessions. The data was first analyzed within each cultural group (1) and then compared among groups for similarities and/or differences (2) as detailed below.

1. The main ideas exposed during each focus group session were identified, and representative quotes were then extracted. The four focus group sessions were then analyzed together, with main concepts across sessions sorted out, summarized, and illustrated with select quotes. The results were summarized according to the question sets used during the focus groups sessions: motherhood, children’s vegetable consumption, influences on children’s vegetable intake, factors involved, and barriers to children’s vegetable consumption (Table 1).
2. To analyze the similarities and differences across cultural groups, the transcripts of the three cultural groups were codified through a coding framework developed based on the research objectives, the script, and the findings obtained in the first analysis (analysis within cultural groups) (Table 3). The coding was conducted with the qualitative data management software QRS NVivo 12 (version 2018, QSR International Ltd., Melbourne, Australia). After sorting responses in each category, quotations that illustrated the main concepts were selected. Additionally, the number of times each coded concept was mentioned by each cultural group was tallied (Table 3). If the same participant repeated the same idea, it was only counted once.

## 3. Results

### 3.1. Within Cultural Groups

#### 3.1.1. Chilean Mothers (CL)

##### Motherhood (CL)

Chilean mothers agreed that becoming a mother had improved their eating habits.

“*Before being a mom, I ate horribly, McDonald’s and other things like that… Since I became a mom, I am more conscious*”.

“*Yes, absolutely (my eating habits have been improved since I become a mother), before I never had the amount of vegetables at home that I have now*”.

Regarding which kind of vegetables were offered to children, the large majority of Chilean mothers stated that they would not offer vegetables that they did not like to their children.

“*I would never think to offer my children something that I do not like… It happened to me once, that we went to a friends’ house and they had grilled bell peppers, and they offered it to me, and I said no, and then they offered it to my son and he said yes, and he ate everything! (laughing) I would have never offered that to him*”.

“*In my case, I hate cauliflower, I would never prepare it, or buy it. But, for instance, broccoli is not something that I like, but I can eat it, so sometimes I prepare it at home*”.

Chilean mothers stated that it is very important for them that their children eat vegetables. Some Chilean mothers even expressed some feeling of guilt if their children rejected vegetables.

“*If my son does not eat vegetables one day, I feel like I am not being a good mom*”.

“*Feeding my children well is part of my role as a mom, if they do not eat well, I feel that I am failing*”.

##### Children’s Vegetable Consumption (CL)

When Chilean mothers were asked about how many vegetables they thought their children should eat, all of them answered with an ideal specific number:

“1/3 *of the plate*”,

“2 *times a day*”,

“*A small bowl of vegetables divided in the different meals of the day*”,

“*I guess fourservings a day*”

Regarding what determined children’s vegetable intake, the majority mentioned sensory and hedonic factors.

“*I feel that vegetables are not to children’s taste… for children everything is very sweet, even toothpaste! Vegetables are not like that*”.

“*I think it is the taste… Vegetables are tasty, but not as tasty as meat, for instance grilled steak! My daughter liked it (steak) immediately*”.

“*My son has a problem with the texture of salad, he can eat other salads like tomatoes, but he has problems with leafy salad*”.

Moreover, in all Chilean focus groups, mothers stated that their children could eat vegetables if they did not see them. Because of this, many Chilean mothers used a stealth strategy of hiding vegetables.

“*They will eat more if I am able to hide the veggies better*”.

In this sense, Chilean mothers stated using different strategies to hide vegetables, from cutting the vegetables very small or grinding them to masking their color with other foods.

“*My children can eat vegetables if they cannot see them, everything (that they eat) is camouflaged. So, when I make pasta, I prepare a lot of sauce in which I include a lot of vegetables …The only veggie that they can see and eat is broccoli. I started grinding everything, then I started chopping everything very small, so they cannot easily separate the vegetables from the rest of the dish. I started since they were small, but I still prepare their food in this way*”.

Some Chilean mothers also talked about the age factor as a determinant of children’s vegetable intake, which related with the possibility of choosing what they ate.

“*Before (when she was younger) she ate more (vegetables)… or at least, she complained less. Maybe it is also, because now she knows that there are options, cereal, cheese, chocolate, and she asks for that. She always asks for a yogurt (before starting to eat a meal) and I give it and then she eats*.”

“*I feel that there is also an age factor. Because before my daughter did not argue (about food), what was on her plate was what she must eat. Now she is super picky, she says ‘Oh no, today I do not want to eat broccoli, I want to eat cheese*”.

##### Influences on Children Vegetable Intake (CL)

The majority of Chilean mothers thought that they, as parent, have an important influence on their children’s vegetable intake.

“*I think parents influence a lot. I have had to rehabilitate my husband; he was the kind of person that eats steak with rice and nothing else. And children do what they see and that’s what they eat*”.

“*I feel that now is my moment. So, I try to be as healthy as possible. Soon the moment will arrive when my children will have two dollars in their pocket, and they will be able to choose to go eat at McDonald’s*”.

“*It depends 100% on me. If I do not offer it to him, he is not going to eat that. Sometimes, there is no time… But ideally, we would be giving him veggies*”.

Some mothers also expressed that peers influence their children’s vegetable intake while others were unsure about it.

“*Now (peers) have a lot of influence. Sometimes, my older daughter arrives home (from school) saying ‘Ew yuck’ and this is something that she hears out…. We also use the influence between children, because my younger daughter eats better, so we try to celebrate it trying to encourage the older one to copy her behavior*”.

“*Honestly, I am not sure how much is peer influence. In my case, my son imitates what another child does, but when he is at home, he does not eat the same, because he does not like it*”.

##### Factors Involved (CL)

Regarding the difficulty of making children eat vegetables, some Chilean mothers stated that the biggest challenge was not so much getting children to eat vegetables but rather having them eat a variety of vegetables.

“*I don’t find it difficult (to make children eat vegetables). What is difficult is that they eat variety. If I give them cucumber or tomato it will work for me perfectly, never so well as if I give them chicken with rice and ketchup, that’s a winner…the difficulty for me is to make them eat more than these two or three vegetables that they like*”.

There was also a group of Chilean mothers who thought that the main challenge for them as parents was “hiding” the vegetables better and having time to prepare them.

“*For me it is a lack of time to prepare vegetables more attractively or finding new preparations in which I can hide them better*”.

Regarding why it is important that children eat vegetables, the majority of Chilean mothers talked about the nutritional value of vegetables.

“*Vitamins, nutrients, and fiber*”.

Some Chilean mothers also spoke about the importance of developing healthy habits and developing a balanced diet.

“*Now they are developing the habits and acquiring the taste for healthy food*”.

##### Barriers to Children’s Vegetable Consumption (CL)

When barriers to children’s vegetable consumption were discussed, many Chilean mothers stated that the food environment is a fundamental factor in the promotion of healthy eating habits. Many of them made comparisons between the Chilean and Californian food environments.

“*It happened to me when I went to Chile to live for a year. Everyone offered my children sweets and sweet drinks. It is super difficult to maintain a healthy diet if the environment does not help. The environment is essential*”.

“*When I went to Chile, my son wanted to drink water, he was used to, and my mother-in-law said no, juice, juice, how are you going to give water to the child*?”.

“*In Chile, when you go to someone’s house, you have to offer them a sweet drink or juice. Offering water looks bad in Chile*”.

Some Chilean mothers mentioned that their problem was the snacking of their children.

“*My child runs to the kitchen the whole day asking for snacks, and I think this means that my son is not hungry at dinner time*”.

Regarding specific recommendations about how to promote vegetable consumption, many Chilean mothers felt that the challenge for them was in improving presentation, the flavor of the dish, and finding better ways to hide vegetable in the dishes

“*I think it is a mixture of being able to make things more attractive and hiding them better*”.

##### Other Items (CL)

Three mothers, participants in two different sessions, spoke about childhood memories regarding of how their parents forced them to eat. Even if these mothers justified the eating practices used on them when they were children, all of them believed that these strategies were traumatic, and they did not repeat them with their own children.

“*(When I was a child) I was a very bad eater and I had a very bad time because of that, because they (my parents) always forced me to eat. And I swore to God that I would never do it like this with my children… The only thing I ate, until I was one year old, was strawberry yogurt, and it was the only thing I ate…Since I was close to malnutrition my mom used to chop the meat very small and put it inside of my strawberry yogurt*”.

“*I don’t know if it happened to you (asking other mothers), but my parents left me sitting until I ate everything. There was nothing about self-regulation or those things. They literally forced me to eat everything (that was on my plate)*”.

“*In my case it was the same, at least when I was very young, I was always forced to eat in a bad way. I am unsure if I really hated the foods, or if the way that my parents fed me made me reject foods*".

#### 3.1.2. Chinese Mothers

##### Motherhood (CN)

Regarding how motherhood had affected Chinese participants’ eating habits, the majority thought that their eating habits had improved.

“*Previously I usually had vegetables in two meals. After having a child, because the family pays attention to the consumption of vegetable, my husband started to cook vegetables for breakfast. At breakfast the portion (of vegetables) is smaller… It is just to tell myself to eat more vegetables, to pay attention to nutritional issues… And it is mainly for the kid*”.

“*I changed my methods after my child was born. I shifted from oily stir fries to boiling vegetables. In the past I only stir fried. I think I learned this from my mom at the beginning, but then I changed for my own family*”.

“*I don’t like beans, but in order to let my children eat beans, I am willing to try with them. So, my liking is not changed, but the habit is somewhat changed*”.

Some Chinese mothers stated that they would not offer something that they did not like, and others stated that they tried to do it with specific vegetables.

“*There are so many different kinds of vegetables, that I prefer to buy and cook the ones that I know and that we (as a family) like better*”.

“*Before being pregnant, I only ate restricted types of vegetables. After my son was born, I started to eat a greater variety of vegetables to develop his interest. Even if some of these vegetables, I do not particularly like*”.

When speaking about the importance of children eating vegetables, everyone agreed that it is very important and many of the Chinese mothers explained that vegetables are an essential part of their eating habits.

“*Vegetables are essential in a meal*”.

##### Children’s Vegetable Consumption (CN)

When the quantity of vegetables children should eat was discussed, Chinese mothers tended not to give an exact amount and to answer based on their child’s body cues.

“*If my son has a normal bowel movement and is not getting sick too often, I will not be worried about his amount of vegetables*”.

“*I think, I don’t pay attention to the absolute amount, and if they don’t eat enough (vegetables) in this meal, for the next meal, for example, I will make more vegetables. I try to balance that*”.

Some mothers also spoke about the percentage of the dish.

“*Usually, we have half a plate of rice and half of the main dish. For the main dish, vegetable occupied 70 percent. Meat occupied the rest*”.

Regarding which factors determine children’s vegetable intake, Chinese mothers talked about sensory characteristics, mood and age factors. The majority of their comments touched on sensory factors somehow.

“*There are some foods that are harder to be accepted by children because they have a special flavor. For example, celery and parsley have a bad aroma. He (my son) didn’t eat carrots in the beginning because carrots have a smell that he did not like, now he eats them because I add them into other dishes…That is why, I do not think that it is about giving what the kid likes but giving the kid everything and he will eat them*”.

“*Children like the food with a colorful appearance and stronger saltiness*”.

“*I think it is a texture thing. My son picks out any tomatoes that appears in a dish, he does not like their texture (the texture of cooked tomato)*”.

“*I realize that most of the food children don’t like are the foods with a lot of fiber. After they grow up, maybe this will change*”.

Some mothers talked about specific mood situation.

“*I think there is a mood factor. If they are sick, they will not eat*”.

Regarding the age factor on children’s vegetable consumption, Chinese mothers thought that it did not have a significant influence.

“*The difference is not big. When they were small, they were not picky. After growing up they started to complain about some foods, but they still ate them*”.

“*My kids don’t have much difference. They will eat anything they have had before and the things which taste good*”.

However, there were some who thought that children ate more when they were younger, and others who believed that children ate more vegetables when they grew up.

“*I think that when they are small, they eat whatever I cook*”.

“*I think they ate less when they were small and where less conscious. I think maybe they ate more meat, so they are too full to eat vegetables. After turning eight years old, they become more mature and know the importance of a balanced diet... I think it depends on the growing stage of the child*”.

##### Influences on Children’s Vegetable Intake (CN)

Regarding how much parents influence children’s eating habits, the majority thought that parental influence is high.

“*I think the influence is large, because when I gave something good to them from when they were young, when they grow up, they like that food. However, if they didn’t try some foods when they were small, they will not like that food later*”.

“*I think the influence is quite large. Because the kid observes and sees what you do. Even if you cook food and then you don’t eat that food, they will not try the dish. Commonly if the parents are not picky, the kids are also not picky. My husband is picky, so my kids are picky, too*”.

Another parent thought that parental influence was important, but not always successful in changing children’s preferences.

“*We can influence, but the influence is not everything. It is very hard to try to change a child’s liking*”.

On the subject of peer influence, Chinese mothers thought that it could influence children’s consumption, but as it is not as constantly as parental influence; they were not sure how much it could influence.

“*If they see their friends eat the food, they will try some. If they don’t like it, then they will not finish the food*”.

##### Factors Involved (CN)

In general, Chinese mothers agreed that if children had the habit and knew that vegetables are part of the meal, they would eat them.

“*I do not have too much of a problem because we always have vegetables for lunch and dinner, so they already know that there will be vegetables to eat, so they eat them*”.

Some Chinese mothers complained about the variety of the vegetables that their children consume.

“*The types (of vegetables that my child eats) are restricted, like carrots, corn and lotus root. And he refuses any leafy greens*”.

“*I think being balanced is very important, they should not eat a restricted and unbalanced diet*”.

Additionally, they agreed that it is easy to give children the vegetables that they like, but difficult to give the ones that they do not like.

“*I think if the vegetables are ones which they like, it is easy, but if it is not what they want, they strongly refuse to eat*”.

When the topic of why it is important for children to eat vegetables was discussed, the majority of Chinese participants talked about concrete health issues, many of them related to digestion.

“*Health, and if my kid doesn’t eat vegetables, his bowel movement is going bad*”.

“*My* *knowledge told me that if I don’t eat enough vegetables, I will have vascular problems and abnormal bowel movements*”.

Some Chinese mothers also mentioned that children’s vegetable consumption is important due to the enjoyment of eating something delicious.

“*To have a balanced and sufficient diet. It could provide healthy nutrition to the kids. But I also think if they eat more vegetables, they could have more delicious food*”.

“*We eat vegetables at home not just because they are healthy, but because we think they are delicious, and our family enjoys eating them*”.

Some Chinese mothers also talked about the nutritional reasons for why eating vegetable is important.

“*Nutritionally* *important, like fiber, because I think the digestive tract needs fiber*”.

“*I* *also care about health. I think eating vegetables not only provides vitamins and fiber but is also natural*”.

##### Barriers to Children’s Vegetable Consumption (CN)

Chinese mothers stated that the main challenge for increasing the vegetables consumption of their children was their cooking skills.

“*It is because my cooking skills are not good enough, so I am not willing to buy and try new foods and new recipes*”.

“*I think it is hard because I don’t cook well. He likes the vegetables provided by the day care center; sometimes I cook the same things, but he dislikes it*”.

Many of them also commented that some vegetables present in the market are unknown to them, which is an obstacle to increasing the variety of vegetables in their cuisine.

“*In the US, I find some new vegetables in the grocery store, but I never have tried them before, so I am afraid to try them, because they are strange, and I do not know how to cook them*”.

Moreover, many of them explained that if they increase the amount of vegetables, they would need to reduce the amount of the other components, like rice and meat, which are also important in their diet.

“*It is because my kids also want to eat some meat*”.

In this regard, about half of the Chinese participants thought that the amount that they currently ate was the right amount.

“*Actually, I think that now we have a diet that is balanced*”.

“*What we eat is enough*”.

Regarding salad, some participants explained that their children did not have the habit of eating salad, because in their culture, salads are not an important part of a dish.

“*We are not interested in salad. We rarely eat salad, because the size and nutritional ratio doesn’t seem worth it. And since I was born and grew up in China, I do not like raw vegetables*”.

Regarding specific recommendations for making children eat more vegetables, some Chinese mothers talked about specific formats of preparations in which vegetables are more accepted by children.

“*He likes dumpling, no matter what vegetables are in it, he always eats all of them. If he eats less of certain food, I put that stuff into a dumpling and he starts to eat*”.

Another mother suggested mixing the vegetables that her children didn’t like with mushrooms. “If there is something they don’t like, I add some mushroom or tree mushroom, which have the flavor they like and they eat it”. 

Other mothers stated that explaining to children the health properties of vegetables is a successful strategy for making their children eat vegetables.

“*For something that they dislike, I will tell them that it is good for their health, so they will eat some*”.

Additionally, hunger was mentioned by some mothers as a strategy of vegetable consumption.

“*If they are hungry, they will be more likely to eat. When he arrives home from school, he generally eats everything*”.

#### 3.1.3. American Mothers 

##### Motherhood (US)

The majority of American mothers tended to think that their eating habits improved since they became mothers.

“*I skipped meals all the time before. Now I cannot*”.

“*I changed a lot. I used to eat a lot of fast food, but now it’s a treat. Going out to dinner was something to do, but with the family, it’s more of a rarity, because we have to balance things in a different way*”.

“*It’s different, now I have family dinners. It’s not the same as when you get home at 8 p.m. and just eat some pasta and ice cream… Now, I would eat more vegetables because I feel more pressured to make sure the kids have it with every meal*”.

American participants agreed that they did not offer their child something that they do not like.

“*I am not going to buy something that I do not like, but if someone offers it to them… (it is fine)*”.

“*I cook for the whole family. I like almost everything, and since I’m the cook, I’m not going to choose something I don’t like*”.

“*I like Brussels sprouts but no one in my house likes them, so I don’t usually get them. My husband loves beets, but I don’t, so I don’t get them neither*”.

“*You have to be prepared to eat it yourself, and I wouldn’t want to eat a vegetable I don’t like*”.

Only one American mother mentioned trying to give food that she did not like to her children.

“*Everything that I like most of the time they would like, but when it comes to dislikes, I am not a bell pepper fan, even in salad, but I make sure they get it. I mean, I do not necessarily want them not to like something because I don’t like it*”.

When American mothers were asked about how important it is for them that their children eat vegetables, many explained that the most important thing for them was to make vegetables available for their children, to give them the possibility to eat them, even if their children sometimes rejected them.

“*Vegetables are really important in the diet. My kid eats some but not all, and I’m not going to get bent out of shape about that. We make vegetables available; we eat a lot. It is important to me, but I am not worried about that*”.

“*She eats everything, but just not vegetables. I am not pushing her. I try to have vegetables around, and I hope she will eat them eventually*”.

##### Children’s Vegetable Consumption (US)

When discussing the amount of vegetables children should eat, some American mothers gave a specific number or amount.

“*Two servings a day*”.

“5 *or whatever is recommended*”.

“*Twice a day*”.

And another group answered what they would like their children to eat.

“*I would be happy with twice (a day) if it was a decent volume each time*”.

“*I’d like to see them eating more vegetables than anything else*”.

On the topic of what determines children’s vegetable intake, American mothers in general talked about the sensory characteristics of vegetables.

“*I think she just likes flavors that are sweet and salty, and vegetables are sometimes bitter. Even if she likes broccoli*”.

“*I think that the flavor doesn’t have a lot that’s appealing to a kid… And the texture*”.

“*Strong flavors are hard for little kids. Cucumber is popular because it tastes like water…like potatoes, everybody likes them*”.

Additionally, a group of mothers talked about age as a determinant of children’s vegetable intake.

“*I feel that he is in a struggle age. I do not know if it is true, but I feel that if I make too much of an issue about eating, it will escalate. I can’t win every power struggle*”.

“*I just have a normally picky 2-year-old, which is funny because at first he would eat anything: avocado, sweet potato, anything. Now it’s not just anything and we have to beg him to eat broccoli and put random things to eat to make him do it. So, it sadly curtails what we eat sometimes because it is wasted otherwise*”.

“*My daughter does not eat vegetables; the only thing that she is able to eat consciously is raw broccoli. I do not want to cook something and hide vegetables, just for her. I am not worried about it; I think it is just her age*”.

##### Influences on Children’s Vegetable Intake (US)

American mothers tended to agree that parents can exert a positive influence, but they did not agree on how much.

“*I feel that exposure is very important, you can like or not like it, but exposure gives kids the opportunity to like foods*”.

“*My kids are older. So, absolutely, I think. What I’ve served them over the years is what they have grown to like. It has influenced their own preferences*”.

However, some American mothers did not feel that they had an influence on their child’s vegetable intake.

“*I like most vegetables, but they have more dislikes. They give me the tomato on the hamburger or whatever. They’re more particular and more disliking and pickier. I do not think that I have a lot of influence on that*”.

Perceived peer influence was mixed.

“*She can try something new if a friend is eating that, even though she will not eat the same at home with me*”.

“*I think if there is any peer influence, it is slightly positive*”.

“*When they were younger, I used to worry about the peer influence being a more negative thing, but now that they are older, I think it is more positive*”.

##### Factors Involved (US)

In general, American mothers agreed that making children eat vegetables was not hard, but what was hard was making them eat a variety of vegetables.

“*The ones that they like, yes are easy. The problem is making them eat more than that*”.

“*It is easy if I give them the vegetables that they like. They eat tons of carrots so easily, but they don’t want to even take a look at green vegetables. It is harder to get them to eat a variety and the ones that are most packed with nutrients*”.

When the importance of eating vegetables was discussed, many American participants gave nutritional and health arguments in support of children’s vegetable consumption.

“*It is very important. There is a huge amount of vitamins and it is low in sugar and high in fiber and minerals. I am concerned about future health problems… I would like to prevent (them) by eating*”.

“*I think it’s important because of health reasons. I have seen too much diabetes in people’s family and hmm… I don’t want to do that. I’m looking more at long-term habits*”.

“*HBO literally say that the children born today are expected to die before their parents because of the diet in America, so that is how important vegetables are*”.

Additionally, two mothers mentioned the importance of eating vegetables in order to maintain a healthy body weight.

“*Because I’ve been away a lot, the only thing I realized is that it is the way to stop my son from being overweight, I have seen my nephews and they don’t eat vegetables. And so, part of what he’s learning when he is young is to eat less of the sweet stuff*”.

There were also opinions regarding the importance of eating vegetables from a social perspective.

“*To be able to go into the world, in the world there is a lot of vegetables, they need to be able to go to restaurants, people’s homes and they cannot always pick out the vegetables. They’re going to be getting a lot of mixed foods—they can’t always be picking certain foods out at fancy restaurants*”.

“*Food is a big part of culture and it’s good to be comfortable with a wide variety of foods, it is very important*”.

##### Barriers to Children’s Vegetable Consumption (US)

For many American mothers, the main obstacle to their children eating more vegetables was the amount of work for them as mothers, including buying, cooking/preparing, and offering vegetables to their children.

“*If you are going to use fresh vegetables, you have to prepare them before they spoil. If someone delivered fresh salad to my door, we would eat it. It is just the amount of effort that goes in*”.

“*It’s the amount of effort that you put in and the results. (Because) I do not know if they (my children) are going to eat them*”.

“*When my husband and I were both working fulltime, time was a big factor. Meat is really easy to cook, and vegetables take a long time to prepare and cook. It is a time intensive thing, so when you are busy and stressed, that can be a factor as well. Planning, grabbing, cutting, preparing*…”.

“*I think it’s more than just my likes and dislikes, because getting them to eat healthy takes extra effort*”.

Some American mothers also brought up the concepts of satiation and craving in the discussion.

“*If we would eat more vegetables, we wouldn’t be as satisfied. Like, I like cheese, meat, and hummus; if I added more vegetables I would not be satisfied*”.

“*It is also a combination between convenience and what your body is craving. Packaged and boxed snacks are easier and that’s what they’re craving. If they eat carrots, they’d have to eat a lot of vegetables to satisfy a craving. It is a more instant solution*”.

Some American mothers advocated for persistence, as in offering children the option to eat vegetables if they wanted.

“*It is more about consistency because they change so frequently. Kids grow so fast, that you have a new child each three months, so if you stop offering, you will kind of miss that window. So, you really have to keep going. It does not matter if there is a right or wrong answer, you had to have keep doing it*”.

Additionally, many mothers talked about gardening and cooking as a way to promote vegetable consumption.

“*We were trying to get our daughter to gain weight, but we could only do that with unhealthy food like ice cream. We tried to incorporate more vegetables. How to do that? So, we started cooking butternut squash and let them watch us cook. We tried to include the kids in the vegetable preparation process. And now we have this side for dinner, so it is improving*”.

“*Gardening and having the child see where it’s coming from is very helpful. He enjoys eating the vegetable much more if he’s the one who picked it. We just changed his school (in this new school they grow vegetables), and now I can see that he is more willing to try these foods*”.

“*I figured out that giving her control gets good results. When we are in a store and I ask her what vegetable, she would like to eat, at home she will be more willing to eat that*”.

Two mothers talked about competing food items on the child’s plate.

“*If there are other things on the plate, they’ll choose that first*”.

### 3.2. Across Cultural Groups 

#### 3.2.1. Major Challenges to Children Eating Vegetables

Thirty-two references to major challenges to the consumption of vegetables by children by the mothers in this study were coded (Table 3). Notably, the difficulty of achieving a variety of vegetable intake in children was mentioned eight times—four times by Chilean mothers, and twice by Chinese mothers and American mothers. The challenge of improving their cooking skills in order to offer more attractive options to children was mentioned eight times—two times by Chilean mothers and six times by Chinese mothers. The challenge of the time that is involved in offering vegetables to children (time to shop, shelf life in the refrigerator, and time to prepare them) was mentioned 10 times—twice by Chilean mothers and eight times by American mothers, but none by Chinese mothers. The presence of other foods that compete with the consumption of vegetables was mentioned six times, four times by Chilean mothers, and twice by American mothers. 

#### 3.2.2. Mother Factors

Ninety-three references were coded in the category “mother factors”. This category includes all the references that were directly related with mothers, among them accepting children’s rejection, feelings of guilt, mothers who do not offer what they do not like, motherhood improving eating habits, and preconceived amounts of children’s consumption (Table 3). Specifically, 32 times mothers stated that their eating habits were improved since they became mothers (mentioned 11 times by Chilean mothers, 10 times by Chinese mothers, and 11 times by American mothers). When we asked how many vegetables children should eat, 32 mothers gave a specific amount, where 14 were Chilean, 15 Americans, and only three were Chinese. Across groups, 18 mothers stated that they did not offer vegetables that they did not like to their children, where five of them were Chilean mothers, four were Chinese mothers, and nine were American mothers. The acceptance of children’s vegetable rejection was stated by seven mothers, all of them American. Finally, there was a group of four mothers, all of them Chilean, who stated that they felt guilty in their role as mother if their children did not eat vegetables. 

#### 3.2.3. What Determines Children’s Vegetable Intake

When the factors involved in children’s vegetable intake were discussed, 103 references were coded for this topic (Table 3). Specifically, 30 references were about parents as important influences of their children’s vegetable intake, 15 by Chilean mothers, 12 by Chinese mothers, and only three by American mothers. Twenty-two references were found about the importance of peer influence on children’s vegetable intake, seven of them in the Chilean group, eight of them in the Chinese group, and seven in the American group. 

Regarding peer influence, the direction of it was also coded, as positive (13 times), negative (four times), or not defined (six times) (Table 4). Child age was also a factor that was mentioned in relation to influence on children’s vegetable intake; 13 references were coded for this item (two by Chilean mothers, three by Chinese mothers, and eight by American mothers).

Finally, the unappealing vegetable flavor for children was mentioned 38 times, 14 by Chilean mothers, 13 by Chinese mothers, and 11 by Americans mothers. According to the analysis of mothers’ perceptions regarding the appeal of vegetable flavor for children, it was mentioned 17 times that the problem was the taste, four times that it was the aroma, 19 times that it was the texture, and five times the appearance (more information on Table 5).

#### 3.2.4. Feeding Strategies

Feeding strategies that were mentioned by mothers to have an effect on their children’s vegetable intake are summarized in Table 3. Hiding vegetables in the food or cutting them so small that children could not separate them from the rest of the dish was a strategy mentioned 13 times (eight times by Chilean mothers, twice by Chinese mothers, and three times by American mothers). Involving children in vegetable gardening and cooking vegetables with them was mentioned seven times, all of them by American mothers. Persistence in offering vegetables (exposing children to vegetables) was mentioned seven times, all of them by American mothers. Allowing children to feel hungry before dinner was a strategy mentioned three times, all of them by Chinese mothers. Forcing children to eat was mentioned seven times, four by Chilean mothers and three by Chinese mothers. Finally, the importance of an environment that promotes vegetable consumption was mentioned four times, all of them by Chilean mothers. 

#### 3.2.5. Why Children Should Eat Vegetables

Finally, 55 references were coded under the reasons why children should eat vegetables (Table 3). The main reason was the health and nutritional benefits of vegetables, which was mentioned 34 times, 10 by Chilean mothers, 14 by Chinese mothers, and 10 by American mothers. The importance of building healthy eating habits in children was mentioned seven times, two of them by Chilean mothers, two by Chinese mothers, and three by American mothers. The importance of following social norms, was mentioned six times, once by Chilean mothers and five times by American mothers. The importance of vegetable intake as part of a balanced diet, was mentioned five times, once by Chilean mothers and four times by Chinese mothers. Finally, the importance of eating vegetables for weight management, was mentioned three times, once by a Chilean mother and two times by American mothers. 

## 4. Discussion

This qualitative research uncovered or confirmed a number of key learnings, which can be acted on to promote the consumption of vegetables in children. Some of these key learnings were consistent across the three ethnic and cultural groups of mothers we studied, and others were more specific to one or two of the groups. Across groups, mothers expressed that their eating habits had improved since they became mothers. Mothers across groups felt that the aim of these changes was to set a better example and to provide a healthier eating environment for their children. Previous studies that have analyzed eating behavior changes during the life span support this finding [32,33,34]. Individuals in life stage transition (e.g., moving in with a partner or the birth of a first child) are more likely to make changes in their nutritional strategies, these important life events being an opportunity for changes toward better food choices [34]. In this regard, studies have observed an association between pregnancy and positive changes in dietary behaviors [32,33]. Regarding vegetable consumption, a significant increase in vegetable consumption in women after becoming mothers has been reported [32,33]. The idea that motherhood is a time of life in which women have the willingness to improve their eating habits is very relevant considering the importance that mothers have in their family’s feeding practices, as well as the direct influence that they exert on their children’s eating behaviors [18,30,35,36]. Moreover, even though our results showed many points of agreement across the cultural groups analyzed in this topic, our results also showed some differences in mothers’ eating habit improvements depending on the cultural group analyzed. In particular, Chilean mothers emphasized increasing the amount and variety of fruit and vegetables eaten and reducing fast-food consumption. Chinese mothers focused their changes on making their cuisine healthier, by reducing the amount of oil and salt used, and American mothers tried to reduce the number of times that they ate out as well as fast-food consumption, and to establish a meal schedule and avoid skipping meals. These different foci represent different nutritional concerns across the cultural groups analyzed, which should be considered when designing nutritional interventions. Moreover, this information reinforces the importance of including mothers in the promotion of children vegetable consumption, taking into account the key role they play in the development of children’s eating behavior [32,33,37], as well as their capacity for improving their own eating habits, in order to provide a better food environment for their children.

The majority of mothers, across cultural groups, expressed that they did not offer foods that they did not like to their children, an idea that it is supported by previous studies [38,39]. The notion that children’s food exposure is conditioned by mothers’ food liking is very relevant, considering the strong influence that food exposure has on the development of children’s food preferences [25,40,41]. Skinner and colleagues (1998) reported a significant correlation between foods never offered to children and their mothers’ food dislikes [42]. Our current study, due to it its qualitative nature, adds to this knowledge by suggesting reasons why mothers show this behavior. Specifically, mothers explained that they would not choose something that they did not like, because they would not know how to cook or prepare it, because it was unpleasant to think of preparing a disliked food, because they as mothers would have to eat that food, and finally because there are so many food options, so why choose a food that they did not like. Moreover, our results show that mothers do not only restrict the food offered to their children based on their own liking, but also based on other family members’, which inevitably adds more restrictions to the food that is offered to the children. In this sense, considering that foods disliked by mothers and other family members will probably not be offered to children, a family with substantial food restrictions will probably reduce the possibility that their children can develop a liking for a varied diet. In this aspect, the literature has consistently shown that the exposition to a variety of different textures, tastes, and aromas during childhood, and especially during early childhood, strongly influences children’s food preferences, likes and, in turn, consumption [14,15,43]. Our results show that a few mothers across groups made an extra effort not to restrict children’s foods exposure based on their own food preferences, being aware that doing so would restrict their children’s food preferences.

As expected, all mothers, independent of their cultural group, indicated that it is important that their children eat vegetables. However, relevant differences among groups were evidenced mainly with regard to the importance they perceived and the responsibility they felt. Specifically, Chilean and Chinese mothers tended to feel that children’s vegetable consumption was mainly their responsibility, whereas for American mothers, vegetable consumption was mainly their children’s own decision, which was linked to their children’s likes and dislikes and their age. In this regard, the literature has reported a connection between specific cognitive stages and an increase of food rejection, consistent with food neophobia [42], which peaks around two years old, an age that was consistently mentioned by American mothers as a period of high rejection. In this sense, previous research has shown that higher levels of food neophobia is related with a less varied diet [44] and with low vegetable intake [45]. Moreover, Johnson (2002) suggested that informing parents about food neophobia as a normal part of children’s development can serve to reduce anxiety and decrease the intensity of power struggles when children reject food [46]. However, it is also important to consider that stereotypes of feeding problems at specific ages (e.g., “terrible twos”) could be overestimated [47]. In this regard, it is possible that the awareness of children’s food rejection, mainly at two years old, expressed by American mothers in our study, could reduce the anxiety of mothers but also predispose them to accept it, instead of encouraging mothers to expose children to a variety of foods. The literature has shown that food neophobia is reduced through exposure and learning mechanisms that ensure that a food is safe to eat [48,49]. Regarding the other two cultural groups analyzed, some Chinese mothers mentioned the age of the child as a possible factor in food rejection, but not in a consistent way, as some stated that children’s age increased vegetable consumption and others felt the opposite way. In the case of the few Chilean mothers who mentioned age as a factor, they believed that it decreased vegetable consumption due to children gaining the ability to ask for, and eat, other foods.

Regarding mothers’ feelings of responsibility and frustration at children’s vegetable rejection, our results show that Chilean mothers tended to feel frustrated and overwhelmed in their role as mothers if their children did not eat the food that they served them. This feeling of guilt or failure of Chilean mothers can be connected to the controlling feeding strategies that many of them mentioned using to ensure children’s vegetable consumption (ground/cut-very-small vegetables). Some Chilean mothers expressly said that they felt they were failing as a mother if their children did not eat well. The idea that food choices can be associated with value judgments regarding the healthiness or unhealthiness of the food eaten by children has being previously reported. Ristovski and colleagues (2010) studied families’ food practices in different cultural groups in Canada and found that mothers tended to associate the idea of being a good mother with having knowledge of nutrition, preparing healthy food, monitoring children’s intake, and using strategies to get their children involved with food [50]. In the case of Chilean mothers, such beliefs could also be rooted in their own experiences as children. Indeed, some Chilean mothers shared memories about their parents using the threat of punishment to force their vegetable consumption. Even though all mothers who shared these memories expressed that these kinds of strategies were unsuccessful and actually traumatic, it may be that the importance of children’s eating habits came from their own experiences as children. In this sense, across cultural groups, Chilean mothers showed more controlling feeding strategies than the other two cultural groups. Regardless, pressuring children to eat reduces vegetable consumption [51], even if this strategy may increase consumption in the short term. Long term, this strategy tends to reduce consumption by reducing the liking of vegetables [48]. American mothers felt that they should provide healthy food to their children and give a good example by eating vegetables. However, they also gave children the responsibility to decide and choose. The American mothers’ awareness of the effect of cognitive stages and personal liking over children’s vegetable consumption could allow them to shed the feeling of absolute responsibility and with it, not to feel overwhelmed if their children decided not to eat vegetables. A completely different interpretation can be made in the case of the Chinese mothers, who did not worry a great deal about their children’s vegetable consumption, even if some mothers in this group would have liked their children to eat more vegetables. This is probably because the average Chinese diet consists of a much higher vegetable consumption, with a recent estimate of an average intake of 350 grams of vegetable a day in China [52], a level of consumption of daily vegetables that comes close to the World Health Organization’s recommended quantity (400 grams/daily) of fruit and vegetables together [53]. Moreover, our results show that Chinese mothers had a very natural relationship with vegetables, expressing that vegetables are a fundamental part of their diet, so even if their children would not eat the expected amount, they were always exposed to them. In this sense, Chinese mothers indicated that if their children did not eat enough in one meal, they would try to balance consumption by preparing more vegetables for the following meal.

Chilean and American mothers showed awareness of dietary guidelines regarding vegetables, and their answers were probably a reflection of what they had heard in vegetable promotion campaigns, yet their own children’s vegetable consumption appeared to fall well below. This was in stark contrast with Chinese mothers, the majority of whom did not answer with a specific value of the amount of vegetables children should eat. Instead, Chinese mothers talked from a nutritional perspective based on their observations of their children’s health. Specifically, Chinese mothers expressed concern about the amount of vegetables consumed by their children if they got sick often and/or started having abnormal bowel movements. The perspective of Chinese mothers on vegetable consumption based on body cues comes from a view in which food is conceived as an essential component of human health. A previous study conducted in England reported that the concept of keeping healthy for the Chinese community required a balanced diet [54]. Moreover, it is also possible that a specific amount is harder to define in a traditional Chinese meal, where dishes are placed in the center of the table and shared by everyone [55], compared to Chilean and American cultures, where everyone eats from their own plate.

The majority of mothers mentioned the sensory characteristics of vegetables as the main determinant of children’s vegetable consumption. In this aspect, the literature has been emphatic in showing the importance of liking on children’s food intake, describing liking as the most important determinant for children’s vegetable consumption [56,57,58]. In the present study, mothers across all three groups expressed that the sensory profiles of vegetables do not fit children’s liking. Chinese mothers talked about the liking of salty tastes that children have, the specific rejection of many textures and the rejection of some strong aromas. Regarding which textures and aromas produce vegetable rejection in Chinese children, there was a disagreement among participants, which could be related to children’s personal liking and to the kind of vegetable and the type of preparation used. Some Chilean mothers also mentioned that young children had problems with some textures, especially related to leafy salads. The majority of Chilean and American mothers mentioned their children’s liking of sweet tastes, which are not commonly present in vegetables. Finally, some American mothers mentioned that their children would reject vegetables with strong flavors. The literature supports the mothers’ opinions regarding the sensory profile of vegetables tending not matching what is innately liked by children. In this sense, vegetables are characterized by having low energy content, often not being sweet, and sometimes being bitter, characteristics that do not match with what is innately liked by humans [13]. In this regard, vegetable preferences need to be learned though experience with food [59]. Salty and sweet tastes are liked by children, while bitter taste is rejected [59]. The avoidance of strong-flavored vegetables mentioned by some mothers could be related to bitterness and sulfur aroma compounds, among others. Similarly, fiber-related textures were often seen as a challenge to liking. In this sense, recipes and preparations that follow children’s vegetable tastes are fundamental. Zeinstra and colleagues (2010) have studied the effect of different preparation methods over children vegetable liking, finding that preparation influences children’s vegetables intake. The preparation methods that children like are also linked to their culture, considering that familiarity is one of the factors that drives children’s vegetable liking [60]. Chinese mothers also stated that mood and hunger affect children’s intake, with bad mood reducing vegetable and general food consumption, while hunger increased it. Hunger is the biological trigger for food intake, and mood can affect food consumption in different ways [61]. However, it is known that children are more likely to eat in an emotionally positive atmosphere [22], which confirms the ideas expressed by Chinese mothers about their children needing to feel well in order to have an appetite.

When the effect of social influence over children’s vegetables consumption was discussed, there was an agreement across cultural groups that mothers could exert an important influence on their children’s vegetable intake. However, while Chilean and Chinese mothers believed in the power of their influence, American mothers tended not to be sure if their influence could produce real changes in their children’s eating habits. The influence of mothers over children’s vegetable intake has previously been analyzed in the literature [26,30,62,63]. Previous studies have shown that mothers exert influences on children’s eating habits though a variety of conscious and unconscious mechanisms, such as exposure, modelling, and feeding practices. These have produced direct effects on children’s vegetable intake [21,23,26,30,62,63]. A mother’s ability to influence a child’s vegetable consumption is exerted across multiple and simultaneous dimensions, with possible positive, negative, or neutral effects on their children’s vegetable consumption. Regarding peer influence, some mothers thought it was important, others were not sure about it. Moreover, among the mothers who thought that peers influenced children’s vegetable consumption, there were some, Chilean mothers in particular, who thought that the influence was positive and other who thought the influence was negative. Some Chinese mothers thought that there was an influence, but not a consistent one, nor one that was able to modify eating behavior. Finally, American mothers tended to think that the influence of peers was slightly positive. A positive modeling peer effect has been observed [64,65], along with a positive effect related to the perceived amount of vegetable intake by peers [66] and a negative influence due to the negative comments that peers make about other children’s lunch in school settings [24]. We have studied children’s vegetable liking resemblance with their mothers’, siblings’, and peers’ and found that culture has an important effect on vegetable liking resemblance of children with their mothers and peers. Specifically, the highest level of agreement was shown for Chinese participants, followed by the US, and finally Chile (the group that showed the lowest level of agreement). Moreover, among all the relationships analyzed (including the three countries), Chilean mothers showed the lowest level of vegetable liking resemblance with their children [67].

Regarding the ease of getting children to eat vegetables, across groups, mothers stated that making children eat vegetables was not very hard if they only offered their children the few vegetables that they liked. In this regard, mothers stated that the harder challenge was to make children eat a variety of vegetables. Across cultural groups there was agreement in the type of vegetables that children tended to like and dislike, dark green vegetables usually being the ones that were mentioned as the most difficult to include in children’s diet. This can be explained by the sensory profile of green dark vegetables, which tend to be bitter, a taste innately rejected by humans [68]. It is also possible to think that mothers tend to offer vegetables that are easily accepted by children (e.g., sweet and starchy vegetables), and rarely offer vegetables that are harder to develop a preference for, making these vegetables less familiar to children. In this sense, the literature has shown that repeated sensory exposure in a positive social context is a successful strategy to increase food preferences [25]. Among the other difficulties that mothers mentioned in getting children to eat vegetables was the time constraint of having perishable foods ready to eat at home (including the time to buy them, washing, cutting, and preparing them), which was expressed mainly by American mothers and also by a couple of Chilean mothers. Previous research has reported vegetable preparation time as an important barrier to increasing consumption [69]. In the Chinese group, some mothers stated that the big difficulties for them were their cooking skills and their ability to prepare more delicious dishes. Some of them even mentioned that for them it was challenging to increase variety because there were some vegetables that they did not recognize at the market, so they were unwilling to buy them, because they would not know how to prepare them. The Chinese group in this study had the constraint of being far away from their family, considering their cultural structure in which grandparents help with meals preparation, but also because they stated that in the US, not all the vegetables that are familiar to them are present, and some of the available ones are completely unknown to them. Even with this constraint, the Chinese participants were the group that expressed fewer problems related to their children’s vegetable intake, which is probably due to their tradition of vegetable consumption.

In relation to the importance of eating vegetables, mothers of all cultural groups indicated the nutritional value of vegetables as one of the main reasons for their consumption. Vegetables are a nutrient dense food, characterized by being an important source of vitamins, minerals, dietary fiber, and antioxidants, all important nutrients in the human diet [70,71]. Mothers in this study showed knowledge and awareness of the nutritional importance of vegetable consumption. The majority of Chilean and American mothers mentioned specific nutritional components (e.g., vitamins, minerals, and fiber) as the main important factors for vegetable consumption. Most Chinese mothers talked about the importance of vegetable consumption as an essential part of human health. Specifically, Chinese mothers expressed that vegetable consumption is important for maintaining general health (e.g., getting sick less often), having normal bowel movement, and cardiovascular protection. Even if American mothers were mainly focused on specific nutritional components of vegetable intake (e.g., vitamins, minerals), some American mothers talked about the importance of their consumption in the prevention of obesity and Type 2 diabetes. Previous research has focused on the positive effects of vegetable consumption on people’s health, showing that vegetable consumption can exert a protective effect against important non-communicable diseases, such as ischemic strokes, obesity, and type 2 diabetes [5,7,8,9,19,71,72]. Some Chinese mothers also talked about vegetable consumption contributing to the enjoyment of a meal, and about the importance of enjoying delicious and nutritious food. The pleasure of eating healthy food can be learned early in life, and is a strong factor in the development of healthy eating habits [73]. 

Regarding the barriers to children’s vegetable consumption, Chilean mothers talked about the importance of the food environment in the development of healthy eating habits. Chilean mothers compared their current location (Davis and Berkeley, California) with the place they were from (Santiago and Concepción, Chile). Specifically, Chilean mothers stated that in Chile, children constantly receive candy from adults (as a reward or to show affection) and that there is heavy consumption of sweetened beverages, instead of water. Both these factors reduce the possibility of developing healthy eating habits. The literature supports the idea that the food environment is key to the development of eating habits, where greater access and availability (either to healthy or unhealthy food) is directly expressed in the community food choices [74]. The arguments given by Chilean mothers are supported by statistics that show that Chile is currently the country with the greatest consumption of sweetened beverages worldwide [75] and that it has the second highest obesity rate of all the countries that belong to the OECD (Organization for Economic Co-operation and Development). In the case of some Chinese mothers, what prevented their children from eating more vegetables was their ability to prepare more tasty dishes (which was also commented on by some Chilean mothers). Researchers have shown that palatability is essential in food acceptance, which is especially important in the case of children, with liking described as the most important determinant for children’s vegetable consumption [56,57]. Many Chinese mothers also stated that their children were eating enough vegetables, and if they ate more, they would need to reduce the amount of other important components in their diet, which is something that they did not want to do. This returns to the idea that Chinese culture values a balance diet and that vegetables are thought of as an essential part of a meal [54,55]. In the case of American mothers, the main constraint was the time needed for mothers to prepare vegetables, which has been supported by previous research as an important limiting factor for vegetable intake in consumers [69]. 

With regard to mothers’ recommendations for promoting vegetable consumption, participants shared strategies that had worked for them. Many Chilean mothers stated that hiding vegetables in food was the best strategy for them, which consisted of cutting vegetables very small or grinding them up in a way that was not noticeable or impossible to separate from the rest of the dish. Moore and colleagues (2007) studied the feeding strategies used by mothers of preschoolers in England, reporting mashing and cutting the food very small as a strategy used by mothers, likely to reduce sensory exposure to the vegetables’ flavors and textures [76]. This is supported by the idea that vegetable preferences need to be learned though positive experiences with food, in which many consecutive sensory experiences may be needed [59]. The strategy that worked for some American mothers was to involve children in cooking and gardening. Different studies support this recommendation by showing the positive effect of involving children in growing vegetables and/or preparing and cooking them. This positive and recreational exposure to vegetables increases the knowledge of them, their familiarity, and also the willingness to taste them, which has been linked to increased liking and intake [5,77,78]. Finally, Chinese mothers’ recommendations were to mix the vegetables that children do not like with very palatable ones, such as mushrooms. The role of associative conditioning has been studied as a strategy to promote vegetable consumption in children, showing that vegetables with liked accompaniments can be a useful initial strategy for children with high levels of food neophobia and low vegetable liking [79]. This strategy seeks to increase initial willingness to taste non-liked vegetables, and through repeated exposure the liking of this vegetable can be increased and with it also its intake [79]. Additionally, some Chinese mothers also recommended hunger as a successful strategy for children’s vegetable consumption. There is no doubt that hunger is the biological activation for food intake [61]. In this sense, too many snacks could be detrimental to children’s vegetable intake at mealtime.

It is important to report some possible limitations of this study. The American group of mothers only included Euro-Americans, because they are the ethnic majority in the community in which we conducted our study. All cultural groups were recruited in two college towns in Northern California (Davis and Berkeley). All participants were living in the US, which could imply some acculturation for the Chilean and Chinese participants. This could mean that Chilean and Chinese participants were more aware of some cultural differences than Americans, and they could have been more critical in their vision than Americans, who did not necessarily have that direct comparison. Moreover, we also think that the recruitment of all cultural groups in the same setting provided some consistency, as the food environment was similar for all participants. It is also worth mentioning that all the participants of this study had higher education, and all of them indicated that they did not have economic restrictions in buying vegetables. Without direct food or environmental restrictions, it is possible to analyze the mothers’ visions of their children’s vegetable consumption and see cultural imprints on their perceptions and attitudes. It is also important to highlight that the results of this study can only be extrapolated to these cultural groups. Finally, as with any qualitative study, additional validation of the key learnings uncovered or confirmed herein through quantitative experimental designs, measures, and analyses would be warranted.

## 5. Conclusions

Without a doubt, mothers are very relevant actors in the development of children’s eating habits. This present study analyzed mothers’ opinions to contribute to the understanding of the development of children’s vegetable preferences and intake. In this sense, this study confirms the influence that mothers have over their children’s eating habits, specifically vegetable intake, which is conditioned by mothers’ experiences with food and by their own cultural life story. Moreover, mothers from different countries have had different experiences with food, and this cultural background plays an important role in the feeding practices that they as mothers use with their children. The analysis of mothers’ opinions regarding the way that they feed their children provided rich information that can be useful when implementing interventions to promote vegetable consumption. Moreover, this study shed light on key information across all cultural groups analyzed. A key finding was that mothers change their habits for health reasons when they get pregnant, information that can be used to encourage pregnant women to improve their eating habits by targeting them with messages about the nutritional value of vegetables. Additionally, the possibility of children developing a varied diet was highly conditioned by the tastes and preferences of mothers and other family members. The sensory characteristics of vegetables were another limiting factor noted by mothers of all cultural groups. Vegetables are "not for children’s tastes", since they are usually not sweet, not salty, and sometimes bitter. This big challenge to promoting vegetable consumption by children can be addressed by working on culinary strategies to modify some of these sensory characteristics, making them more suitable to children’s tastes. Moreover, these possible strategies must be targeted to children’s realities, especially in regard to age and culture. Interventions aimed at increasing children’s vegetable consumption need to be designed and implemented at a local level in order to take into account the cultural factor, which is key in the understanding of human relationship with food. Finally, this study uncovered some successful strategies used by mothers to increase vegetable consumption by children.

## Figures and Tables

**Table 1:** Focus group script.

Greetings	
Outline of study purpose	
Instruction for participants	
Explanation of research subject rights	
Ice breaker and introduction of the participants	
Warm-up questions	
Question set 1: Motherhood	
	*"Did you change your eating habits since you became a mother?"*
	*“Do you offer your child vegetables (and foods in general) that you do not like?”*
	*“How important is to you that your child eats vegetables?"*
Question set 2: Children’s vegetable consumption	
	*"How many vegetables do you think a child should eat?"*
	*"What determines children’s vegetable intake?”*
Question set 3: Influences on children’s vegetable intake	
	*"How much do you think that peers influence your child’s food preferences?"*
	*"How much do you think that you as a parent influence your child’s food preferences?"*
Question set 4: Factors involved	
	*"Is it easy/hard to get children to eat vegetables?" Why is it easy/hard?*
	*"Why is it important that your children eat vegetables?"*
Question set 5: Barriers to children’s vegetable consumption	
	*"What prevents you and your children from eating more vegetables?"*
	*“Do you have any recommendations for making children eat more vegetables?”*
Dismissal and thanks	

**Table 2:** Participants’ characteristics *.

	Chileans (*n* = 19)	Chinese (*n* = 19)	Americans (*n* = 20)
Educational level			
Graduate or professional degree	89.4%	89.4%	55%
Bachelor’s degree	5.3%	5.3%	35%
Some college	5.3%	5.3%	10%
Occupation			
Salaried or hourly position	36.8%	57.9%	55%
Graduate student	0%	0%	5%
Stay at home parent	57.9%	42.1%	35%
Business owner	5.3%	0%	5%
Marital Status			
Married or living with a partner	100%	100%	80%
Divorced or separated	0%	0%	20%
Number of years living in the USA			
5 years or less	74%	74%	NA
More than 6 years	26%	26%	NA
Child age			
2 to 4 years old	48.1%	33.3%	29.6%
5 to 7 years old	29.6%	33.3%	26%
8 to 12 years old	22.2%	33.3%	44.4%
Economical restriction to buy vegetables			
	0%	0%	0%

NA = not applicable; * This table was built using the demographic information provided by mothers in an exit survey at the end of the focus group sessions.

**Table 3:** Comparison of concepts mentioned by mothers between cultural groups.

	Total *n* of References	N.R.CL	N.R.CN	N.R.USA
Major challenges				
Archive variety	8	4	2	2
Cooking skills	8	2	6	0
Time consuming	10	2	0	8
Presence of other foods (e.g., snacks)	6	4	0	2
Mother factors				
Accepts rejection	7	0	0	7
Feeling of guilt	4	4	0	0
Mothers do not offer what they do not like *	18	5	4	9
Motherhood improves eating habits *	32	11	10	11
Preconceived amount of children’s consumption *	32	14	3	15
What determines children’s vegetable intake *				
Vegetables’ sensory profile	38	14	13	11
Age	13	2	3	8
Parental influence *	30	15	12	3
Peer influence *	22	7	8	7
Feeding strategies *				
Gardening and cooking	7	0	0	7
Hiding vegetables	13	8	2	3
Hunger	3	0	3	0
Persistence	6	0	0	6
Forcing children to eat	7	4	3	0
Promoting environment	4	4	0	0
Why children should eat vegetables *				
Balanced diet	5	1	4	0
Building healthy eating habits	7	2	2	3
Health and N = nutritional benefits	34	10	14	10
Social norms	6	1	0	5
Weight	3	1	0	2

N.R. = Number of times that each concept was mentioned as a challenge in children’s vegetable intake by each ethnic group (N.R.CL: Number of references Chile; N.R.CN: Number of references China; N.R.USA: Number of references the United Stated of America). Asterix (*) represent the ideas that were asked about directly, these questions were: Do you offer to your child vegetables that you do not like? Did you change your eating habits since you became mother? How many vegetables do you think a child should eat? How much do you think that you as parent influence your child’s food preferences? How much do you think that peers influence your child’s food preferences? Do you have any recommendations to make children eat more vegetables? Why is it important that your children eat vegetables?

**Table 4:** Reference frequency of peer influence over children vegetable consumption.

Peer Influence	Total *n* of References	N.R. CL	N.R. CN	N.R. US
+	13	2	6	5
−	4	2	1	1
indeterminate	5	3	1	1

N.R. = Number of times that each concept was mentioned as a challenge in children’s vegetable intake by each ethnic group. (N.R.CL: Number of references Chile; N.R.CN: Number of references China; N.R.USA: Number of references the United Stated of America).

**Table 5:** Reference frequency by sensory dimension that contribute to children vegetable rejection.

Sensory Dimensions	Total *n* of References	N.R. CL	N.R. CN	N.R. USA
Taste	17	7	5	5
Aroma	4	1	1	2
Texture	19	5	8	6
Appearance	5	3	2	0

N.R. = Number of times that each concept was mentioned as a challenge in children vegetable intake by each ethnic group. (N.R.CL: Number of references Chile; N.R.CN: Number of references China; N.R.USA: Number of references the United Stated of America). * Some mothers mentioned more than one sensory dimension (appearance, aroma, taste, and texture), as sensory factors that produce vegetable rejections by children.
